# Complete Genome Sequence of the *Streptococcus gallolyticus* subsp. *gallolyticus* Strain DSM 16831

**DOI:** 10.1128/genomeA.00108-17

**Published:** 2017-04-20

**Authors:** Imke Grimm, Jessika Dumke, Tanja Vollmer, Dennis Hinse, Christian Rückert, Jörn Kalinowski, Cornelius Knabbe, Jens Dreier

**Affiliations:** a Institut für Laboratoriums und Transfusionsmedizin, Herz und Diabeteszentrum Nordrhein-Westfalen, Universitätsklinikum der Ruhr-Universität Bochum, Bad Oeynhausen, Germany; b Institute for Genome Research and Systems Biology, Center for Biotechnology, Universität Bielefeld, Bielefeld, Germany

## Abstract

*Streptococcus gallolyticus* subsp. *gallolyticus* DSM 16831 is an intriguing strain because of its low virulent phenotype compared to other isolates. We present here the complete genome sequence for this strain isolated from koala feces.

## GENOME ANNOUNCEMENT

*Streptococcus gallolyticus* subsp. *gallolyticus* (formerly *S. bovis* biotype I) is a commensal of the human or animal gastrointestinal tract. In addition, it is a pathogen causing different diseases, especially infective endocarditis, septicemia, and meningitis (1–4). The *S. gallolyticus* subsp. *gallolyticus* strain DSM 16831, isolated from koala feces, is characterized by low virulence and shows additional strain-dependent features in different assays. This strain is the only one of 23 tested strains which is unable to invade endothelial cells, is more rapidly killed in macrophages, adheres just marginally to collagen, leads to fast cytokine expression in whole blood, and is more susceptible to lysozyme compared to other tested strains of this species (5) (I. Grimm, M. Weinstock, I. Birschmann, J. Dreier, C. Knabbe, T. Vollmer, submitted for publication; I. Grimm, N. Garben, J. Dreier, C. Knabbe, T. Vollmer, submitted for publication). To distinguish better between infection-associated *S. gallolyticus* subsp. *gallolyticus* strains [e.g., BAA-2069 (6)] and this low virulent strain, the genome of DSM 16831 was sequenced and analyzed.

The *S. gallolyticus* subsp. *gallolyticus* strain DSM 16831 grew overnight in 30 mL BHI (Oxoid, United Kingdom) and genomic DNA was extracted by the phenol-chloroform method. The genomic DNA was sequenced with Nextera technology and 2 × 250 nt long reads on a MiSeq machine (Illumina, USA). The genome was sequenced to a 113-fold coverage and assembled using Newbler v2.6. Genome finishing was performed using the software Consed (7) and Sanger reads were used to generate a complete genome sequence. Annotation was performed by GenDB v2.4 (8). Phage regions were identified by PHAST, inserting elements by ICEberg, virulence genes by VFDB (virulence factors of pathogenic bacteria), and resistance genes by RGI (resistance gene identifier) (9–12).

The genome has a length of 2,492,900 bp with a G+C content of 37.7%. Annotation disclosed 2,396 coding sequences, 12 tRNAs, and 18 rRNAs. Interestingly, the genome of DSM 16831 contains many integrative and conjugative elements with a total of 13 transposases. Additionally, three regions in the chromosomal DNA of DSM 16831 consist of phage-associated genes. Thereby, one of these regions is complete (integrase *BTR42_02375*, terminase *BTR42_02525*, phage structure proteins, and proteases) and has high similarity with the streptococcal phage P9 (NC_009819). In addition, sequence analysis revealed 11 possible genes involved in antibiotic resistance. Six of these genes code for efflux pumps, two for beta-lactam resistance or mupirocin, aminocoumarin, and fluoroquinolone resistance proteins. Possible virulence genes code for agglutinin receptors (e.g., *BTR42_07910*), trigger factors (e.g., *tig*), different secretion systems (e.g., *essC*), or fibronectin-binding proteins (e.g., *fbpA*). In contrast, DSM 16831 lacks the virulence-associated genes *pil1* and *pil3*, which explains to some extent the low virulent phenotype of this strain (13, 14).

The complete sequence of the genome of DSM 16831 is a valuable tool for analyzing genetic principles of the pathogen *S. gallolyticus* subsp. *gallolyticus* in future studies.

### Accession number(s).

The complete genome sequences of the chromosome have been deposited in DDBJ/EMBL/GenBank under the accession no. CP018822.
